# *Strongyloides stercoralis* Infestation in a Child: How a Nematode Can Affect Gut Microbiota

**DOI:** 10.3390/ijms22042131

**Published:** 2021-02-21

**Authors:** Stefania Pane, Anna Sacco, Andrea Iorio, Lorenza Romani, Lorenza Putignani

**Affiliations:** 1 Department of Diagnostic and Laboratory Medicine, Unit of Parasitology, Bambino Gesù Children’s Hospital, IRCCS, 00165 Rome, Italy; stefania.pane@opbg.net (S.P.); anna.sacco@opbg.net (A.S.); 2 Department of Diagnostic and Laboratory Medicine, Unit of Parasitology and Multimodal Laboratory Medicine Research Area, Unit of Human Microbiome, Bambino Gesù Children’s Hospital, IRCCS, 00165 Rome, Italy; andrea.iorio@opbg.net; 3 Immunology and Infectious Diseases Unit, University Department of Pediatrics, Bambino Gesù Children’s Hospital, IRCCS, 00165 Rome, Italy; lorenza.romani@opbg.net

**Keywords:** Strongyloidiasis, *Strongyloides stercoralis*, gut microbiota, targeted-metagenomics, host-parasite-microbiota interaction

## Abstract

*Background:* Strongyloidiasis is a neglected tropical disease caused by the intestinal nematode *Strongyloides stercoralis* and characterized by gastrointestinal and pulmonary involvement. We report a pediatric case of strongyloidiasis to underline the response of the host microbiota to the perturbation induced by the nematode. *Methods:* We performed a 16S rRNA-metagenomic analysis of the gut microbiota of a 7-year-old female during and after *S. stercolaris* infection, investigating three time-point of stool samples’ ecology: T_0_- during parasite infection, T_1_- a month after parasite infection, and T_2_- two months after parasite infection. Targeted-metagenomics were used to investigate ecology and to predict the functional pathways of the gut microbiota. *Results:* an increase in the alpha-diversity indices in T_0_-T_1_ samples was observed compared to T_2_ and healthy controls (CTRLs). Beta-diversity analysis showed a shift in the relative abundance of specific gut bacterial species from T_0_ to T_2_ samples. Moreover, the functional prediction of the targeted-metagenomics profiles suggested an enrichment of microbial glycan and carbohydrate metabolisms in the T_0_ sample compared with CTRLs. *Conclusions:* The herein report reinforces the literature suggestion of a putative direct or immune-mediated ability of *S. stercolaris* to promote the increase in bacterial diversity.

## 1. Introduction

Strongyloidiasis is considered a neglected tropical disease caused by the intestinal nematode *Strongyloides stercoralis* and characterized by gastrointestinal (GI) and/or pulmonary involvement, with an estimated global prevalence of about 350 million cases [1]. It is endemic in the tropical and subtropical regions of the world where human wastes contaminate the environment, but it is widespread in Europe and in hypo-endemic areas in Italy [2,3]. Once the larvae penetrate the skin, they reach the bloodstream and invade the lungs’ alveoli and this pulmonary migration may cause pneumonia, but usually in an asymptomatic way. The larvae are then expectorated, traveling through the trachea and then swallowed. The larvae mature and become adult parthenogenetic females, which release eggs into the GI tract. The eggs hatch while still in the GI tract and give rise to rhabditoid larvae, which are excreted. However, some of these larvae become infectious (filarioid) and penetrate the anal mucosa and perianal skin, re-entering the circulatory system and restarting the cycle. Because of this auto-infection cycle, a person can be infected with *S. stercoralis* for decades [4].

In immunocompetent subjects, the infection is usually asymptomatic, with low minimal and intermittent larval excretion and may bring a combination of uncertain clinical symptoms such as severe epigastric pain, chronic diarrhea, constipation, indigestion, anorexia, anal pruritus, abdominal distension, weight loss, nausea, vomiting, peripheral eosinophilia, asthenia, adynamia, fever, hemorrhage, anemia, and, rarely, obstruction of the small intestine [5,6].

However, in some predisposing conditions, such as initiation of immunosuppressive therapy and kidney transplant recipients, the disease may change to any form of hyper-infection or disseminated types of strongyloidosis [7].

Herein, we report a pediatric case of *S. stercoralis* hyper-infection, for which larval forms were characterized in stool samples of an asymptomatic patient with a nephrotic syndrome. The parasitological diagnosis was based on light microscopy, molecular PCR-based investigation, and serology. Coupled with parasitological exams, a stool time-point sampling underwent to assess microbiota modulation in terms of ecology and global composition, comparing the patient’s gut microbiota during the infection, post-infection, and using reference, age-matched healthy subjects as controls (CTRLs). This study was conducted to describe the response of the host microbiota to the perturbations induced by the nematode pathogen.

## 2. Results

### 2.1. Morphology-Based and PCR-Based Methods for the Identification of S. stercoralis

**Figure 1:**
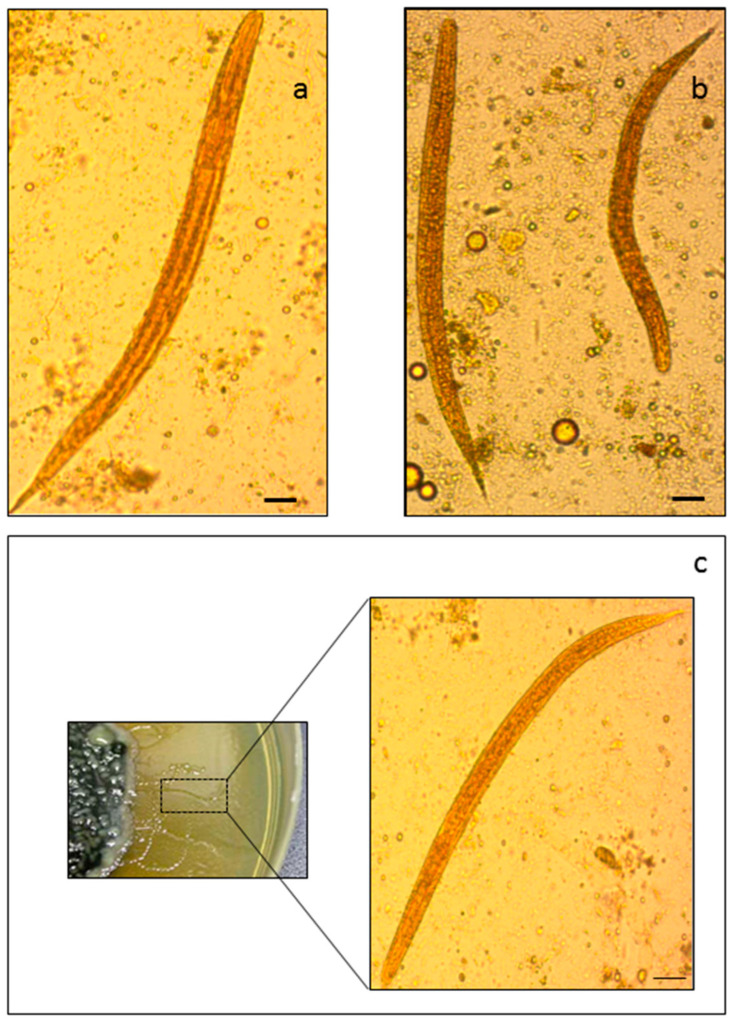
Light microscope-based characterization of *Strongyloides stercoralis*. Rhabditiform larvae of *S. stercoralis* are reported in panels (**a**–**c**) at 40× magnification. The inset refers to a larva onto an agar plate.

By examining stools under light microscopy, a large number of rhabditiform larvae of *S. stercoralis* were identified. The identification of larvae (L3) of *S. stercoralis* was based on nematode morphological features: (i) long esophagus, with a visible bowel junction, (ii) pointed tail, and (iii) mouthparts. Rhabditiform larvae were 250–280 mm in length, with a relatively short, but pointed tail, and a buccal cavity (Figure 1, panels A and B).

The agar plate culture technique was conducted on positive stool specimens and, after seven days, the sealed plates were examined under the 20× microscope objective and motile larvae actively moving were observed under a 40× objective (Figure 1, panel C).

Moreover, DNA amplification of *S. stercoralis* was successfully obtained in this positive stool sample using species-specific primers, thus, confirming the microscopic examination (data not shown).

According to the microscopic and molecular PCR-based investigation, patient serology also resulted in a positive. Instead, microscopic examination of the multiple bronchial washings and CSF samples appeared negative.

### 2.2. Gut Microbiota Profiling Associated with S. stercoralis Infection

#### 2.2.1. Shaping of Patient’s Gut Microbiota Ecology

We monitored the patient’s gut microbiota shaping since the nematode infection to the second month of negative stool samples, relying on three time-points of stool sampling (T_0_: during parasite infection, T_1_: a month after parasite infection, and T_2_: two months after parasite infection). A total of 1,718,328 high-quality reads were obtained from the T_0_, T_1_, and T_2_ samples, and 16 stool samples from healthy, age-matched individuals, with a mean of 85,916 high-quality reads per sample, were used as a gut microbiota reference for comparisons.

The number of OTUs detected at each time-point sample ranged from 1365 to 790, highlighting a decrease of the gut microbiota diversity from parasite infection (T_0_) to post-infection (T_2_) time points (Table 1).

**Figure 2:**
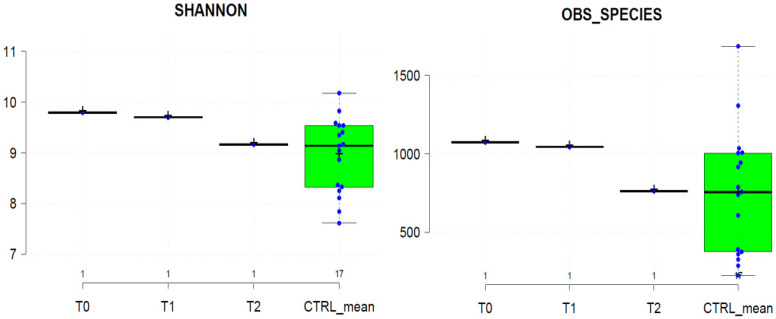
Alpha-diversity metrics of patient’s gut microbiota at T_0_, T_1_, T_2_, and healthy CTRL group (CTRL_mean). Left and right panels show Shannon and Observed_Species diversity indexes, respectively. Center lines show median values and box limits indicate the 25th and 75th percentiles.

This decrease of the gut microbiota diversity from T_0_ to T_2_ was also confirmed by the calculation of the alpha-biodiversity indices. As shown in Figure 2, the T_0_, T_1_, and T_2_ alpha-diversity indices are included in the variability range of the healthy controls. However, T_0_ and T_1_ samples showed higher alpha-diversity values than the T_2_ sample and the average of control groups.

**Figure 3:**
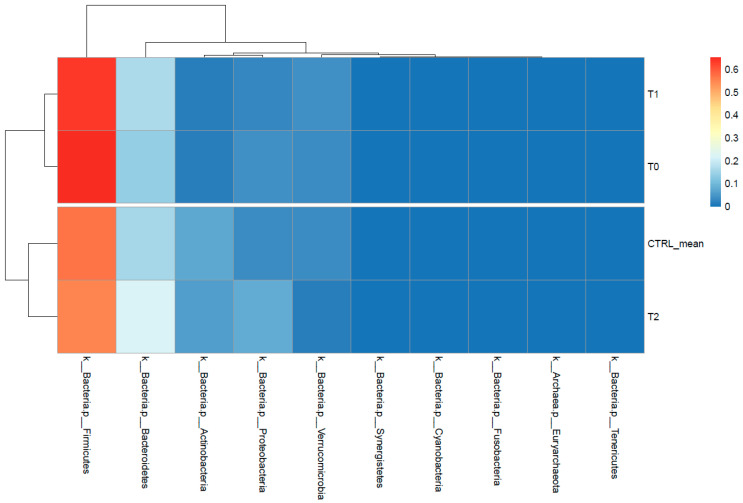
Heatmap with hierarchical clustering based on phyla (L2) abundances. The colors in the heatmap refer to the phylum’s abundance, according to the color scale on the right.

A diverse range of bacterial phyla (L2) were identified in stool samples from the patient and healthy controls, including Bacteroidetes, Firmicutes, Proteobacteria, Verrucomicrobia, and Actinobacteria. In the gut microbiota from controls, Firmicutes were mostly prevalent (57.00%), followed by Bacteroidetes (15.90%), Actinobacteria (7.49%), Proteobacteria (3.86%), Verrucomicrobia (3.66%), and others (12.09%). Compared to CTRLs, the relative proportions of these phyla were mostly maintained in the T_2_ sample. Instead, a clear shift in phyla proportions was observed in the T_0_ and T_1_ samples. Particularly, the T_0_ and T_1_ samples were depleted in Actinobacteria and enriched in Firmicutes with respect to the T_2_ sample and CTRLs, as reported in the heatmap with hierarchical clustering of Figure 3.

**Figure 4:**
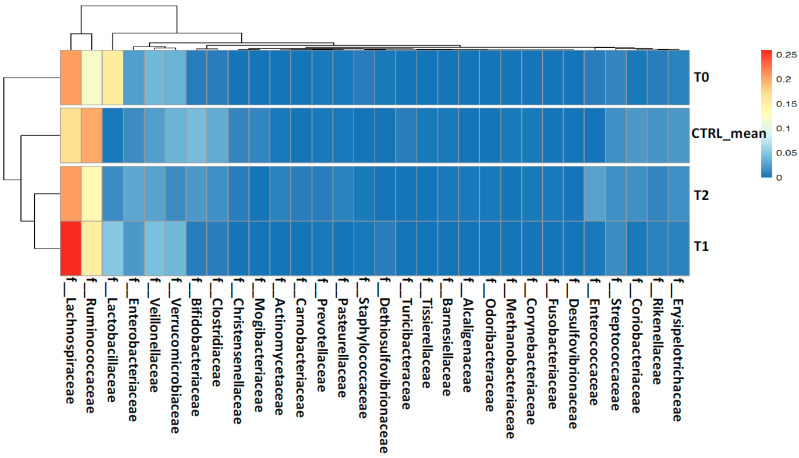
Heatmap with hierarchical clustering based on families’ (L5) abundances. The colors in the heatmap refer to the family’s abundance, according to the color scale on the right.

**Figure 5:**
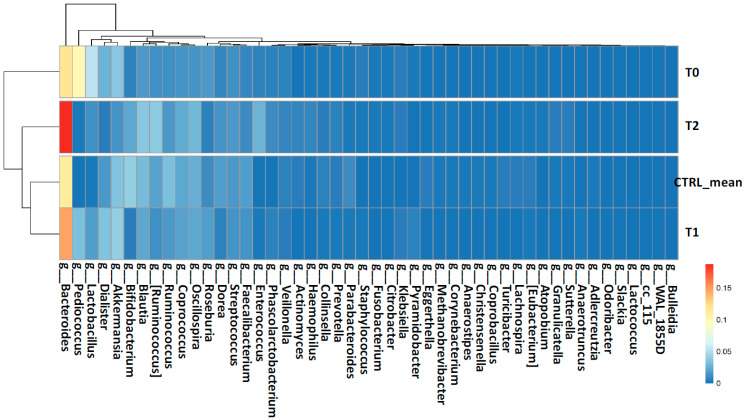
Heatmap with hierarchical clustering based on genera (L6) abundances. The colors in the heatmap refer to the genus abundance, according to the color scale on the right.

At family (L5) and genus (L6) levels, the proportion of several bacterial families and genera were mostly similar between the T_1_ and T_2_ samples by clustering with the CTRLs, while the T_0_ sample showed a different and specific gut microbiota pattern (Figure 4 and Figure 5). In particular, at family level (L5), Bifidobacteriaceae, Coriobacteriaceae, Rikenellaceae, Clostridiaceae, Erysipelotrichaceae, and Ruminococcaceae, showed a gradual enrichment from the T_0_ to the T_2_ time-points. Meanwhile, Veillonellaceae, Staphylococcaceae, Lactobacillaceae, and Lachnospiraceae displayed a gradual depletion from the T_0_ to the T_2_ samples (Figure 4).

At genus level (L6), *Bifidobacterium*, *Blautia*, *Ruminococcus*, *Bacteroides*, *Corynebacterium*, *Colinsella*, *Streptococcus*, *Coprococcus*, and *Oscillospora* showed a gradual enrichment from the T_0_ to T_2_ time-points. Instead, *Staphylococcus*, *Lactobacillus*, and *Pediococcus* displayed a gradual depletion from the T_0_ to T_2_ samples (Figure 5).

**Figure 6:**
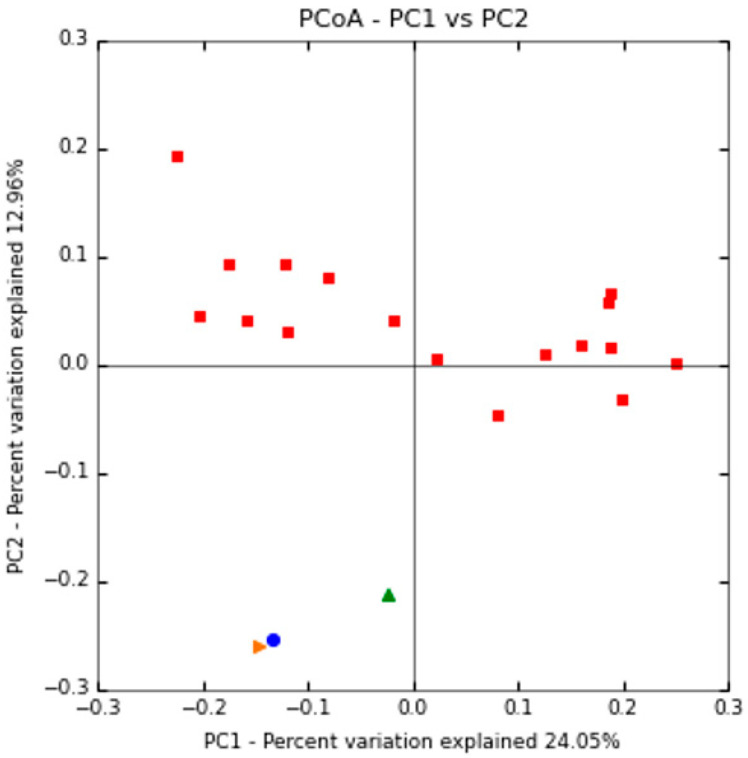
PCoA of beta-diversity values based on Bray Curtis distances. Patient gut microbiota at T_0_: blue circle, T_1_: orange triangle, T_2_: green triangle, and healthy controls: red squares.

The gut microbiota biodiversity between the three patient’s time-point samples and CTRLs was also analyzed via beta-diversity. According to the previous results highlighted by hierarchical clustering, this analysis revealed a shaping of the patient’s gut microbiota during (T_0_) and after (T_1_ and T_2_) nematode infection, with the T_1_ sample close to the T_0_ and the T_2_ sample shifted to the controls (Figure 6).

#### 2.2.2. Metabolic Prediction of the Patient’s Gut Microbiota during *S. stercoralis* Infection

To correlate the fecal microbiota composition data and inferred changes in bacterial metabolism with the response to the parasitic helminth infection, we conducted a predictive metagenomics analysis using PICRUSt. Functional prediction suggested that there were differences in the bacterial functional content of the T_0_ gut microbiota with respect to the healthy controls.

**Figure 7:**
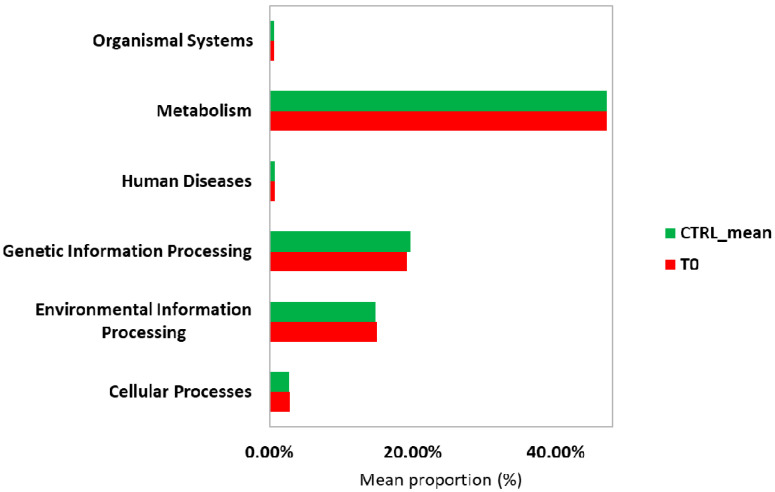
Predicted functional pathways (KEGG Level I) of the gut microbiota during infection (T_0_) with *S. stercolaris*.

**Figure 8:**
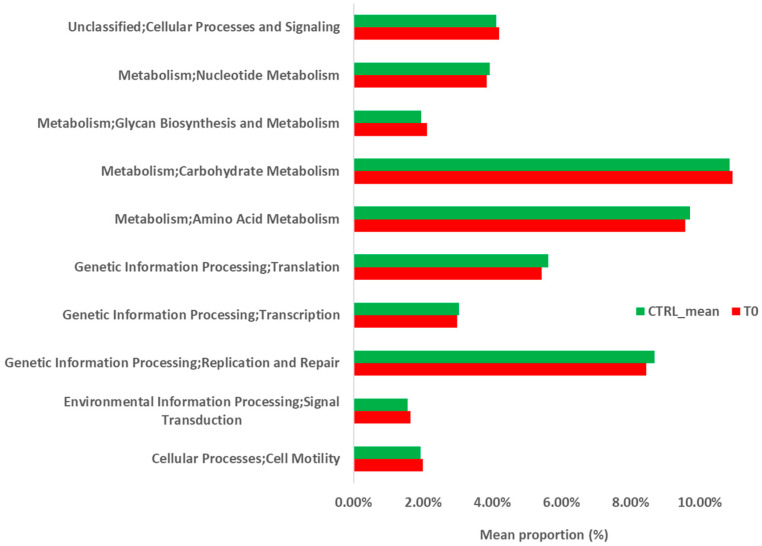
Predicted functional pathways (KEGG Level II) of the gut microbiota during infection (T_0_) with *S. stercolaris*.

**Figure 9:**
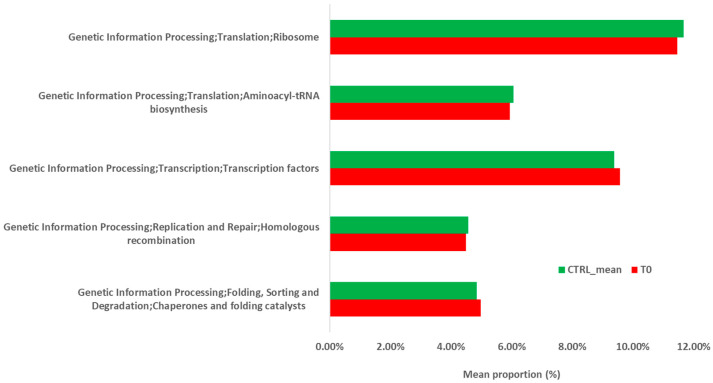
Predicted functional pathways (KEGG Level III) of the gut microbiota during infection (T_0_) with *S. stercolaris*.

At KEGG level I (Figure 7), “Genetic Information Processing” pathway was depleted in the T_0_ sample with respect to the controls and, for this reason, the prediction analysis of this pathway was further deepened by KEGG level II and III. At KEGG level II (Figure 8), “Cellular processes and Signaling,” “Glycan Biosynthesis and Metabolism,” “Carbohydrate Metabolism,” “Signal Transduction,” and “Cell Motility” pathways were enriched in the T_0_ sample with respect to the controls. “Nucleotide Metabolism,” “Amino Acid Metabolism,” “Translation,” “Transcription,” and “Replication and Repair” were depleted in the T_0_ sample when compared with the controls. At KEGG level III (Figure 9), “Transcription factors” and “Chaperons and folding catalysts” pathways were enriched in the T_0_ sample with respect to the controls. Meanwhile, “Ribosome and Aminoacyl tRNA biosynthesis” and “Homologous recombination” pathways were depleted in the T_0_ sample with respect to the controls.

## 3. Discussion

In this report, we performed a targeted-metagenomic analysis of the gut microbiota of a seven-year-old female during and after *S. stercolaris* infection to improve our understanding of how the parasitic infection may influence the host’s gut microbiota.

The patient was admitted to the Academic Department of Nephrology of the Bambino Gesù Children’s Hospital for a nephrotic syndrome with several symptoms, including visual hallucinations, abdominal pain, respiratory stress, and widespread skin rash. After negative radiological exams, clinicians suspected a parasitic infection on the basis of the patient’s origin and her systemic and respiratory involvements.

Several microscopic, serological, and molecular approaches were used to discern, characterize, and identify larvae in the patient’s stool samples and to confirm the diagnosis. The laboratory diagnosis of strongyloidiasis was made by reporting rhabditiform larvae in the stool samples of the pediatric patient, even if in the absence of eosinophilia. However, several reports highlight that eosinophilia can be considered as a nonspecific marker for the screening of chronic strongyloidiasis, especially for individuals without severe gastrointestinal symptoms [8]. After diagnosis of strongyloidiasis, the patient received albendazole treatment for about three weeks (one day after the T_0_ sampling point). The anti-helmintic treatment (albendazole) resulted in the resolution of the infection and in an improvement of the overall patient’s health.

Regardless of the overall similarities in the composition of the patient’s gut microbiota during and after parasitic infection with respect to the healthy controls at a phylum level (L2), our analysis revealed differences in the bacterial profiles of the three time-point samples (T_0_, T_1_, and T_2_), thus, indicating that *S*. *stercoralis* infection was associated with shifts in the relative abundance of specific gut bacterial species. The alpha-diversity indices were higher in the T_0_ and T_1_ samples when compared to the T_2_ sample and the average of the healthy controls. According to previous investigations, increased levels of the bacterial alpha-diversity have been reported for the gut microbiota of individuals infected by several GI helminths (i.e., *Necator americanus*, *Trichuris trichiura*, and *Ascaris* spp.) [9]. Since alpha-diversity indices are used as a proxy of the microbiota “health” (high alpha-diversity is generally associated with a stable and healthy gut bacterial environment [10]), several authors proposed that the direct or immune-mediated ability of GI helminths to promote the increase in bacterial richness and evenness may represent a therapeutic strategy in patients with chronic inflammatory disorders [11].

The differences between the patient’s three time-point samples and healthy controls were displayed by dissimilarities in the relative abundance of particular bacterial taxa in the gut microbiota profiles. As shown by the beta-diversity analysis, the T_1_ sample is very close to the T_0_ sample, while T_2_ was shifted to the healthy controls. These findings suggest that anti-helminthic treatment with albendazole between T_0_ and T_1_ sampling points does not affect the gut microbiota composition. Accordingly, a study on Indonesian treated and untreated subjects with albendazole for helminthes infections showed that this anti-helminthic drug does not influence the composition of the gut microbiome [12].

Our results showed an enrichment of *Bifidobacterium*, *Blautia*, *Ruminococcus*, *Bacteroides*, *Corynebacterium*, *Colinsella*, *Streptococcus*, *Coprococcus*, and *Oscillospora* genera from the T_0_ to T_2_ time-points. Instead, *Staphylococcus*, *Lactobacillus*, and *Pediococcus* displayed a gradual depletion from the T_0_ to T_2_ samples.

Regarding the depletion of *Bacteroides* during *S. stercolaris* infection, one study showed that helminths infection protects mice deficient in the Crohn’s disease susceptibility *Nod2* gene from intestinal abnormalities by inhibiting colonization of inflammatory *Bacteroides* species [13]. Resistance to *Bacteroides* colonization was dependent on type 2 immunity, which promoted the establishment of a protective microbiota. Bacteroidetes exhibit a crucial role in the metabolism of a wide range of carbohydrates [14]. In anaerobic environments, the products of fermentation of these substrates are short-chain fatty acids (SCFAs) that can act as a source of ATP by the host cells [15]. Additionally, SCFAs interact with the host immune system by targeting G protein coupled receptors on intestinal epithelial cells and leukocytes and modulating their development, survival, and function [16]. However, a recent study on the intestinal nematode *Trichinella spiralis* showed that *Bacteroides* genus displayed increased abundances in the *T. spiralis* positive stool samples when compared with the negative samples [17]. Therefore, these conflicting results highlight the need for further investigations in this area and that *Bacteroides* abundance during infection might depend on the parasitic species and/or on the type of host immune response.

According to our results, species belonging to the family Lactobacillaceae, which are capable of triggering host regulatory responses [18], have been widely reported to increase in abundance during helminth infection, irrespective of helminth or host species [19,20]. Moreover, several studies have demonstrated that the high abundance of *Lactobacillus* can enhance the persistence of helminth infection, providing evidence for a mutualistic relationship between helminths and *Lactobacillus* species [21,22].

We also highlighted the depletion of *Ruminococcus* genus during *S. stercoraris* infection. Accordingly, a study conducted by a shotgun metagenomics approach showed that the intestinal helminth *Trichuris suis* has an effect on the gut microbiota of pigs with a significant decrease in *Ruminococcus* [23].

Functional prediction of the bacterial metagenomic profile suggested that there was an enrichment of the “Glycan Biosynthesis” and “Carbohydrate Metabolisms” pathways during *S. stercoralis* infection (T_0_ sample) when compared to the healthy controls. In particular, anaerobic metabolism of non-digestible carbohydrates by the gut bacteria produces short chain fatty acids (SCFAs). SCFAs, such as acetate, propionate, and butyrate, mainly in the lumen, are assumed to interact in terms of antioxidant activity, avoiding anti-inflammatory effects on the intestinal mucosa. Moreover, butyrate and propionate can regulate intestinal physiology and immune function, whereas acetate acts as a substrate for lipogenesis and gluconeogenesis [24]. Bacteroidetes and Firmicutes are the most abundant phyla in the human gut, with Bacteroidetes mainly producing acetate and propionate, while Firmicutes mostly produces butyrate [25]. Therefore, the observed enrichment of the “Carbohydrate Metabolism” pathway during parasitic infection, albeit with a low abundance of *Bacteroides*, may be explained by the high abundance of Firmicutes at the T_0_ sampling point. Therefore, upregulation of this pathway in the gut microbiota may represent a response to oxidative stress in the host intestinal environment during *S. stercoralis* infection.

In summary, this study provides a view of changes in the gut microbiota during the course of a parasitic nematode infection. Our observational time-series experiments explored the pediatric patient’s gut microbiota to elucidate the influence of parasite-related modifications and host metabolic responses to microbiota dynamics in the host intestine.

Although our findings add valuable knowledge to this emerging area of the host-parasite-microbiota interactions, agnostic multiomics-based investigations in experimental models of infection and diseases are mandatory to shed light on the contribution of the parasite-associated modifications in the gut microbiome and on the therapeutic properties of parasitic helminthes.

## 4. Case Presentation and Laboratory Diagnosis

### 4.1. Patient’s Characteristics

A seven-year-old female child was admitted to the Academic Department of Nephrology of the Bambino Gesù Children’s Hospital for nephrotic syndrome. The patient was born in Bolivia but adopted and transferred to Italy since May 2018. Upon admission, blood laboratory results showed haemoglobin 12.9 g/dL (11.1–14.8 g/dL), white blood cells, and full blood count within normal ranges (in particular, eosinophil count was 0.04 10^3^/μL), while C-reactive protein was 4 mg/L (<0.50 mg/L). Her medical history included febrile episode, visual hallucinations, abdominal pain, respiratory stress, and widespread skin rash. Epidemiological and clinical data, including the systemic and respiratory involvements, suggested a parasitic infection. Hence, a collection of multiple stool samples for parasitological and gut microbiota profiling investigations was performed at three different time-points: T_0_- during parasite infection, T_1_- a month after parasite infection, and T_2_- two months after parasite infection. Moreover, multiple bronchial washings and cerebrospinal fluid (CSF) samples were collected to verify the presence of parasites in other body districts.

One day after the T_0_ sampling point, the patient received the anti-elminthic albendazole for two weeks. Therefore, at the T_1_ point, the treatment was already ended two weeks earlier and, at T_2_, it was ended a month and a half earlier.

This study was carried out in accordance with the recommendations of the OPBG Ethics Committee (Protocol No. 1113_OPBG_2016) and was approved on 21 April, 2016. Written informed consent was obtained from the patient and from healthy age-matched individuals, whose faecal samples were available at the BBMRI Biobank of Human Microbiome of the Bambino Gesù Children’s Hospital.

### 4.2. Laboratory Diagnosis of Strongyloidiasis

#### 4.2.1. Optical Microscopy

The laboratory diagnosis of strongyloidiasis was made by optical microscopy characterization of larvae in a fecal specimen. With this purpose, fecal samples were concentrated by an ethyl acetate-based technique and examined under light microscopy at 20× and 40× magnification. The assessment of the morphological characteristics of the larvae was made on a smear of feces stained by Lugol independently by two parasitologists.

#### 4.2.2. Stool Agar Culture

Stool samples were also tested for direct parasite searching through the culture onto *S. stercoralis* Agar (Biolife Italiana s.r.l., Milano). The agar plate culture was performed using approximately 3–5 g of feces and the plates were sealed with adhesive tape to prevent larvae from crawling out of the plate and were incubated at 30 °C for at least 7 days [26].

#### 4.2.3. Immunological Methods

Qualitative detection of IgG to *S. stercoralis* was performed using an enzyme-linked immunosorbent assay based on micro-wells coated with *Strongyloides* antigen (ELISA, S. ratti Bordier Products, Effegiemme).

#### 4.2.4. DNA Extraction and PCR Amplification for *S. stercoralis* Detection

Genomic DNA was extracted from stool by using a QIAamp Fast DNA Stool Mini Kit (Qiagen, Germany) with a slight method modification based on the addition of 1 mL of Inhibit EX buffer (Qiagen, Germany) to the sample heated at 70 °C for 5 min in order to increase the quality of extracted DNA. The DNA was finally eluted with 200 μL ATE buffer. The PCR reactions were performed using the following reaction mixture: 22 µL master mix, (Faststart HIFI PCR SYST.DNTP 500U SIGMA-ALDRICH S.r.L.) 1 µL of each primer (forward: 5′ ATC GTG TCG GTG GAT CAT TC 3′, reverse: 5′ CTA TTA GCG CCA TTT GCA TTC 3′), 3 µL of DNA template, and ultra-pure bi-distilled H_2_O up to a final volume of 30 µL under the following conditions: 1 cycle at 95 °C for 5 min (time-delay), 30 cycle at 94 °C for 30s (denaturation), 58 °C for 45 s (annealing), and 72 °C for 45 s (extension), which is followed by a final extension for 5 min [27]. DNA extracted from stool samples of pediatric patients infected with filariform larvae was used as positive controls, while DNA extracted from microscopically negative samples and ultra-pure bi-distilled H_2_O was used as a negative control of the PCR reaction.

### 4.3. Gut Microbiota Profiling by 16S rRNA Targeted-Metagenomics Sequencing

#### 4.3.1. Bacterial DNA Extraction from Stool Samples

Stool samples were pre-treated by a bead-beating process using 0.1-mm glass beads. This step is crucial because it enables the mechanical disruption of bacteria otherwise difficult to lyse using chemical/enzymatic buffers.

Subsequently, DNA from stool samples was extracted using QIAmp Fast DNA Stool mini kit (Qiagen, Hilden, Germany), according to the manufacturer’s instructions. Sixteen stool samples from healthy, age-matched individuals were used as a gut microbiota reference for comparisons. We checked the purity of extracted DNA by Nanodrop measurements (Thermo Fisher Scientific, Waltham, MA, USA). Samples that failed quality control (DNA yield and purity not adequate for libraries preparation) were re-extracted. Amplification of the variable V3–V4 regions from the 16S rRNA bacterial gene (∼460 bp) was carried out using the primer pairs 16S_F 5′-(TCG TCG GCA GCG TCA GAT GTG TAT AAG AGA CAG CCT ACG GGN GGC WGC AG)-3′ and 16S_R 5′-(GTC TCG TGG GCT CGG AGA TGT GTA TAA GAG ACA GGA CTA CHV GGG TAT CTA ATC C)-3′ as described in the MiSeq rRNA Amplicon Sequencing protocol (Illumina, San Diego, CA, USA). The polymerase chain reaction (PCR) was set up by using the 2× KAPA Hifi HotStart ready Mix kit (KAPA Biosystems Inc., Wilmington, MA, USA). DNA amplicons were cleaned-up by the CleanNGS kit beads (CleanNA, Coenecoop 75, 2741 PH, Waddinxveen, The Netherlands). A second amplification step was performed to obtain a unique combination of dual Illumina Nextera XT indices and adaptor primers. The final library was cleaned-up using CleanNGS kit beads, quantified using Quant-iT PicoGreen dsDNA Assay Kit (Thermo Fisher Scientific, Waltham, MA, USA), normalized and diluted to equimolar concentrations (4 nmol/L). The size of the libraries (600–630 bp) was checked using 2100 Bioanalyzer Desktop System (Agilent Technologies Inc., Santa Clara, CA, USA). Pooled and denatured libraries were sequenced by a MiSeq Reagent Kit v2 (300 cycles) (Illumina Inc., San Diego, CA, USA) on the MiSeqDX Instrument (Illumina Inc., San Diego, CA, USA).

#### 4.3.2. Bioinformatics Analysis

The Illumina sequence raw data were processed using QIIME version 1.9.1 [28]. The workflow started with joined paired-end reads, quality filtering, and library splitting, followed by the detection of the chimeric sequences with the UCHIME algorithm, included in the free version of USEARCH61, and the subsequent removal prior to further analysis.

Cleaned reads were clustered and assigned to operational taxonomic units (OTUs) against the Greengenes 13.8 database [29] with a 97% identity threshold using an open-reference OTU-picking protocol with “usearch”. OTUs were then further normalized using metagenome Seq’s CSS (cumulative sum scaling) transformation and, finally, taxa below a minimum fractional count of 0.01% were filtered from the OTU table.

To highlight differences between the patient’s three time-points’ samples and the controls in term of bacteria abundances, we performed several heatmaps with hierarchical clustering (clustering metric: “correlation” and method: “complete”) in R v.4.0.2 environment using Pheatmap package (https://cran.r-project.org/web/packages/pheatmap/pheatmap.pdf (accessed on 20 February 2021)).

Alpha-diversity was estimated by calculating the Shannon and Observed species indices. Beta-diversity was assessed by calculating the Bray-Curtis dissimilarity index. Differences in community composition were visualized by performing principle coordinate analysis (PCoAs). The molecular functions of the bacterial communities were predicted using the Phylogenetic Investigation of Communities by Reconstruction of Unobserved States (PICRUSt) [30] based on 16S rRNA metadata with the Kyoto encyclopedia of genes and genomes (KEGG) database and Greengenes 13.8 reference taxonomy [31].

## Figures and Tables

**Table 1:** Summary of the analyzed samples for the gut microbiota profiling and sequencing output.

SAMPLE_ID	STATUS	Age	Total Reads Count (N)	Assigned Reads (N)	OTUs (N)
T_0_	During parasite infection	7	53,476	36,643	1365
T_1_	1 month after parasite infection	7	16,830	14,598	917
T_2_	2 months after parasite infection	7	44,876	42,857	790
N-05-1	healthy control	7–8	58,775	57,578	1062
N-05-2	healthy control	7–8	68,144	61,598	1475
N-05-3	healthy control	7–8	54,796	52,930	799
N-05-4	healthy control	7–8	164,725	159,772	1044
N-05-5	healthy control	7–8	84,616	80,331	645
N-05-6	healthy control	7–8	293,539	285,209	978
N-05-7	healthy control	7–8	29,696	29,454	377
N-05-8	healthy control	7–8	40,400	37,790	875
N-05-9	healthy control	7–8	76,140	75,780	463
N-06-1	healthy control	7–8	60,591	60,217	397
N-06-2	healthy control	7–8	46,304	46,014	455
N-06-3	healthy control	7–8	400,539	393,198	1647
N-06-4	healthy control	7–8	69,989	69,092	385
N-06-5	healthy control	7–8	75,864	72,235	781
N-06-6	healthy control	7–8	40,312	39,751	932
N-06-7	healthy control	7–8	9379	9302	320
N-06-8	healthy control	7–8	29,337	26,571	494

## Data Availability

The data that support the findings of this study are available on request from the corresponding author.
